# Cost-effectiveness of trifluridine/tipiracil against nivolumab for heavily pretreated metastatic gastric cancer in Japan

**DOI:** 10.1093/jjco/hyab086

**Published:** 2021-06-14

**Authors:** Yusuke Takushima, Ataru Igarashi, Hiroshi Yoshihara, Kohei Shitara, Toshihiko Doi

**Affiliations:** Department of Health Economics and Outcomes Research, Graduate School of Pharmaceutical Sciences, The University of Tokyo, 7-3-1 Hongo, Bunkyo-ku, Tokyo 113-0033, Japan; Medical Affairs Department, Taiho Pharmaceutical Co., Ltd, 1-27 Kandanishiki-cho, Chiyoda-ku, Tokyo 101-8444, Japan; Department of Health Economics and Outcomes Research, Graduate School of Pharmaceutical Sciences, The University of Tokyo, 7-3-1 Hongo, Bunkyo-ku, Tokyo 113-0033, Japan; Unit of Public Health and Preventive Medicine, Yokohama City University School of Medicine, 3-9 Fukuura, Kanazawa-ku, Yokohama 236-0004, Kanagawa Japan; Department of Health Economics and Outcomes Research, Graduate School of Pharmaceutical Sciences, The University of Tokyo, 7-3-1 Hongo, Bunkyo-ku, Tokyo 113-0033, Japan; Department of Gastrointestinal Oncology, National Cancer Center Hospital East, 6-5-1 Kashiwanoha, Kashiwa, Chiba 277-8577, Japan; Department of Experimental Therapeutics, National Cancer Center Hospital East, 6-5-1 Kashiwanoha, Kashiwa, Chiba 277-8577, Japan

**Keywords:** trifluridine/tipiracil, TAS-102, nivolumab, cost-effectiveness analysis, stomach neoplasm

## Abstract

**Objective:**

Nivolumab and trifluridine/tipiracil have significantly improved the overall survival of patients with heavily pretreated metastatic gastric cancer in different placebo-controlled phase III trials. Accordingly, nivolumab and trifluridine/tipiracil have been approved and recommended for patients with heavily pretreated metastatic gastric cancer in Japan. The aim of this study was to assess the cost-effectiveness of trifluridine/tipiracil against nivolumab.

**Methods:**

A partitioned survival model, which consisted of three health states, namely, ‘pre-progression,’ ‘post-progression,’ and ‘death,’ was constructed. Efficacy and safety data were derived from the TAGS and ATTRACTION-2 trials. Costs were estimated based on the standard clinical pathway and national insurance fee schedules. One-way and probabilistic sensitivity analyses were performed. The threshold value was set to JPY 7 500 000 (USD 68 182) for each quality-adjusted life-year.

**Results:**

The expected median overall survival and progression-free survival were 5.59 and 1.99 months for trifluridine/tipiracil and 5.26 and 1.55 months for nivolumab, respectively. The quality-adjusted life-year and expected costs per patient were 0.4379 and JPY 2 054 625 (USD 18 678) for trifluridine/tipiracil and 0.5295 and JPY 5 018 148 (USD 45 620) for nivolumab, respectively. The expected median progression-free survival and overall survival were equivalent with trifluridine/tipiracil and nivolumab, whereas the expected quality-adjusted life-year with trifluridine/tipiracil was slightly lower than that with nivolumab. However, trifluridine/tipiracil reduced the total treatment cost by JPY 2 963 523 (USD 26 996) compared with that of nivolumab. The incremental cost-effectiveness ratio of nivolumab versus trifluridine/tipiracil was JPY 32 352 489 (USD 294 113) per quality-adjusted life-year gained.

**Conclusions:**

Trifluridine/tipiracil was more cost-effective than nivolumab for patients with heavily pretreated metastatic gastric cancer.

## Teaser

Trifluridine/tipiracil was more cost-effective than nivolumab for patients with heavily pretreated metastatic gastric cancer from the perspective of the Japanese public health care payers.

## Introduction

Gastric cancer is the fifth most common cancer worldwide and the third leading cause of cancer-related deaths (1). The expected outcomes are poor for patients with unresectable metastatic gastric cancer, and treatment is generally limited to palliative chemotherapy. Combination chemotherapy, consisting of fluoropyrimidine-based drugs (5-fluorouracil, S-1 or capecitabine) and platinum-based drugs (cisplatin or oxaliplatin), has been recommended as the first-line therapy strategy for metastatic gastric cancer by the Japanese Gastric Cancer Association (JGCA), the European Society for Medical Oncology (ESMO) and the National Comprehensive Cancer Network (2–4). Furthermore, combination therapy with fluoropyrimidine-based drugs, platinum-based drugs and trastuzumab is used for human epidermal growth factor receptor 2 (HER2; also known as ERBB2)-positive metastatic gastric cancer (2–4). In case of failure of the first-line chemotherapy strategy, combination therapy with paclitaxel and ramucirumab has been recommended as a second-line treatment for metastatic gastric cancer (2–4). In case of failure of both first- and second-line treatments, no global standard third-line therapy strategy has been recommended for metastatic gastric cancer, and treatment methods vary among different countries.

Nivolumab, a fully human IgG4 monoclonal antibody inhibitor of programmed death-1, has significantly improved the overall survival (OS) of patients with heavily pretreated metastatic gastric cancer in the Asian phase III ATTRACTION-2 trial (5). Hence, nivolumab has been recommended by JGCA guidelines and is widely used in Japan to treat metastatic gastric cancer. The 2-year follow-up data of the ATTRACTION-2 trial were published in the proceedings of the ESMO 2018 Congress (6), and the median OS was reported to be 5.26 months [95% confidence interval (CI), 4.60–6.37 months] with nivolumab and 4.14 months (95% CI, 3.42–4.86 months) with placebo [hazard ratio (HR) = 0.62; 95% CI, 0.51–0.76; *P* < 0.0001].

Trifluridine/tipiracil (FTD/TPI, also known as TAS-102) is a novel oral cytotoxic chemotherapy combination consisting of a thymidine-based nucleoside analog (FTD) and a thymidine phosphorylase inhibitor (TPI). FTD/TPI has a unique mechanism of action wherein FTD is incorporated into DNA, resulting in DNA dysfunction (7–9). After the efficacy and safety of the drug were established in the phase III RECOURSE trial (10), FTD/TPI was approved in Japan, the European Union (EU), the USA and other countries as a treatment strategy for patients with metastatic colorectal cancer that is refractory to standard therapies. Furthermore, FTD/TPI has revealed a promising efficacy and acceptable tolerability in patients with heavily pretreated metastatic gastric cancer in the global phase III TAGS trial (11), with a median OS of 5.7 months (95% CI, 4.8–6.2 months) in the FTD/TPI group and 3.6 months (95% CI, 3.1–4.1 months) in the placebo group (HR = 0.69; 95% CI, 0.56–0.85; one-sided *P* = 0.00029). In 2019, FTD/TPI was approved in Japan, the US and the EU for patients with heavily pretreated metastatic gastric cancer and has recently been included in JGCA guidelines, with similar recommendations as those for nivolumab. Only nivolumab and FTD/TPI are listed at the same evidence level.

In addition to efficacy and safety, efficiency and cost-effectiveness have emerged as important factors that impact the choice of drug treatment, particularly for anticancer drugs. Governments, health care providers and the public have displayed an increasing interest in learning more about efficiency in resource allocation. In terms of the economic evaluation of FTD/TPI, several studies have assessed the cost-effectiveness of FTD/TPI treatment for patients with gastrointestinal cancer (12–15). However, few studies have focused on gastric cancer, and none has compared the cost-effectiveness of FTD/TPI and nivolumab. Therefore, in this study, we compared the cost-effectiveness of FTD/TPI and nivolumab for patients with heavily pretreated metastatic gastric cancer from the perspective of the Japanese public health care payer.

## Materials and methods

### Model structure

**Figure 1.**
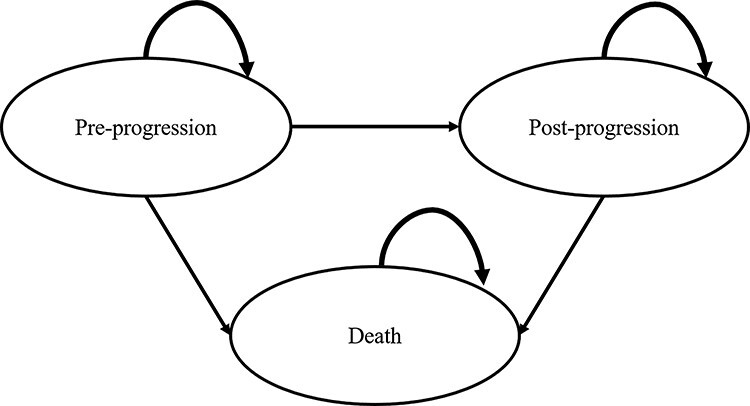
Model structure.

A partitioned survival model (PSM) was generated using TreeAge Pro 2018 R2.1 (TreeAge Software, Inc., Williamstown, MA, USA). PSMs are commonly used for the health–economic analysis of anticancer medications and submission of official dossiers for national health technology assessment agencies, such as the National Institute for Health and Care Excellence (NICE) in the UK (16). In the PSM used in this study, the following three health states were considered: ‘pre-progression,’ ‘post-progression,’ and ‘death’. The PSM revealed the proportion of patients in each cycle, and the cost, life years and quality-adjusted life-years (QALYs) were estimated. The model structure is presented in Fig. 1.

### Treatment protocol

Nivolumab was administered intravenously every 2 weeks, whereas FTD/TPI was administered orally on days 1–5 and 8–12 of each 4-week treatment cycle. Thus, for this analysis, 2 weeks were set as one cycle, and 52 weeks (26 cycles) were considered to be 1 year. The time horizon was set at 520 weeks (10 years). A 10-year period was considered sufficient for lifetime assessment; the likelihood that target patients or those with heavily pretreated metastatic gastric cancer would survive for 520 weeks or longer was considered to be low. In our analysis, death occurred in 99% of patients by 160 weeks on FTD/TPI therapy and by 192 weeks on nivolumab therapy.

### Clinical estimates

Efficacy and safety data were derived from the TAGS and ATTRACTION-2 trials. The probability distribution was applied and estimated in accordance with the most recently published data on OS and progression-free survival (PFS) curves for the FTD/TPI group in the TAGS trial (11) and the nivolumab group in the ATTRACTION-2 trial (6). Superposition of the Weibull distribution was adopted for this analysis as the most appropriate distribution in accordance with the *R*-squared value and Akaike information criterion (AIC). The distribution of each curve was estimated on the basis of the OS and PFS curves using the Weibull distribution. The parameters of each distribution were set to ensure the most appropriate *R*-squared value and AIC figures as follows:

$$ \mathrm{S}\left(\mathrm{x}\right)=w\ {e}^{-{\left(x/\eta 1\right)}^{m1}}+\left(1-w\right){e}^{-{\left(x/\eta 2\right)}^{m2}} $$

Our survival function is a linear combination of two Weibull distributions. Each Weibull distribution has two parameters: *η* and *m*. *m* (>0) is the shape parameter, which affects the shape of the distribution, whereas *η* (>0) is the scale parameter, which stretches or shrinks the distribution. Weights of each distribution are defined as *w* and (1 − *w*), whereas 0 ≤ *w* ≤ 1.

The estimated parameters for FTD/TPI and nivolumab are displayed in Table 1. To validate our model, the estimation was carried out using SAS software version 9.4 (SAS Institute, Inc., Cary, NC, USA). Similarly, OS and PFS distributions were observed for FTD/TPI and nivolumab (data not shown). Similar estimates were implemented for the placebo groups in the TAGS and ATTRACTION-2 trials, and similar results were obtained (*R*^2^ = 0.99967). Therefore, FTD/TPI and nivolumab were comparable in this analysis without any statistical adjustment.

**Table 1:** Parameters estimated for each distribution

Parameter	FTD/TPI	Nivolumab
Overall survival		
*w*	0.230855949	0.368655975
*η1*	33.89876532	7.285138986
*m1*	1.335055548	2.129129407
*η2*	12.193089	28.1395343
*m2*	1.373256081	1.164429921
Progression-free survival		
*w*	0.634034131	0.391491811
*η1*	9.672317513	2.974408627
*m1*	1.383143905	15.91853832
*η2*	3.747731973	11.53140838
*m2*	15.15906897	0.847673601

Our survival function is a linear combination of two Weibull distributions. Each Weibull distribution has two parameters: *η* and *m*. *m* (>0) is the shape parameter, which affects the shape of the distribution, while *η* (>0) is the scale parameter, which stretches or shrinks the distribution. Weights of each distribution are defined as *w* and (1 − *w*), while 0 ≤ *w* ≤ 1.

FTD/TPI, trifluridine/tipiracil.

### Quality of life score estimates

No quality of life (QoL) score data were collected in the TAGS and ATTRACTION-2 trials; thus, for this analysis, EQ-5D data collected in the INTEGRATE trial, a randomized, placebo-controlled phase II trial of regorafenib for the treatment of metastatic gastric cancer (17,18), were derived. The patient population of the INTEGRATE trial had recurrent or metastatic disease that was refractory to two or fewer lines of chemotherapy (including prior treatment with fluoropyrimidine and platinum), similar to the population examined in our analysis. The pre- and post-progression QoL score were set at 0.73 and 0.69, respectively, based on the results of the INTEGRATE trial. As a decrease of QoL score owing to adverse events was not reported for Japanese patients, it was not considered in this analysis.

### Cost estimates

The following direct medical costs were determined and considered in this analysis: costs of drugs, medications, imaging, treatment for adverse events, drugs used after treatment and end-of-life care (Table 2). The drug and imaging costs and medical care fees for the analysis were based on the prices in the national drug tariff and national medical care fee schedule as of 1 December 2018. No confidential discount system has been implemented in Japan. Although drug prices are revised every 2 years, they are fixed in Japan. The willingness-to-pay (WTP) threshold (under which a drug is considered cost-effective) is JPY 7 500 000 (USD 68 182; exchange rate of USD 1 = JPY 110 as of December 2018) for an anticancer drug.

**Table 2:** Model parameters

Parameter	Type of distribution	FTD/TPI	Nivolumab	Range
Cost (JPY)				
Drug price (per 2 weeks)	Gamma	99 969	410 580	±10%
Adverse events	Gamma	17 183.40	6071.10	±25%
Administration (first cycle)	−	47 255.70	47 745.70	−
Administration (after second cycle)	−	5180.00	10 660.00	−
Post-treatment	−	410 580.00	199 938.00	−
End-of-life	Gamma	1 120 000		±25%
Imaging	−	15 200		−
QoL score				
Pre-progression	Normal	0.73		±25%
Post-progression	Normal	0.69		±25%
Others (%)				
Post-treatment transition rate	Triangular	25		0–61
Discount rate	−	2		0–4

FTD/TPI, trifluridine/tipiracil; JPY, Japanese yen.

**Table 3:** Base-case results

Parameter	FTD/TPI	Nivolumab
Cost/patient (JPY)	2 054 625	5 018 148
∆Cost/patient (JPY)	−2 963 523	
QALY	0.4379	0.5295
∆QALY	−0.0916	
ICER (∆cost/∆QALY)	32 352 489	
Median life-years (years)	0.47	0.44
Median life-years (months)	5.59	5.26
∆Median life-years (months)	+0.33	
Median PFS (years)	0.17	0.13
Median PFS (months)	1.99	1.55
∆Median PFS (months)	+0.44	

FTD/TPI, trifluridine/tipiracil; ICER, incremental cost-effectiveness ratio; JPY, Japanese yen; PFS, progression-free survival; QALY, quality-adjusted life-year.

The cost of each drug was determined based on the following body parameters: height, 165 cm; weight, 70 kg and body surface area, 1.73 m^2^. FTD/TPI was administered at a dose of 35 mg/m^2^ twice daily on days 1–5 and 8–12 of each 28-day treatment cycle. The cost for the 4-week therapy was JPY 199 938 (USD 1818); thus, the cost for 2 weeks of treatment was JPY 99 969 (USD 909). Nivolumab was administered at 240 mg every 2 weeks and entailed a 2-week treatment cost of JPY 410 580 (USD 3733).

Not all patients with heavily pretreated metastatic gastric cancer received post-treatment chemotherapy. The post-treatment transition rate was set at 25% in accordance with the results of the TAGS trial. From a conservative perspective, as the cost of nivolumab was higher than that of FTD/TPI, the patients who received FTD/TPI received nivolumab as post-treatment, and the patients who received nivolumab received FTD/TPI as post-treatment. The treatment duration was further shortened in the post-treatment stage; therefore, we decided to implement only one cycle of chemotherapy.

Imaging analyses were conducted every 8 weeks in the TAGS trial and every 6 weeks in the ATTRACTION-2 trial. To ensure that the expenses did not differ between the groups, we decided to perform imaging tests every 8 weeks (based on the opinion of a medical expert) and to conduct these tests conservatively. In clinical practice, imaging tests are conducted when patients receive chemotherapy; however, in this study, the cost of imaging tests during post-treatment chemotherapy was not considered because post-treatment chemotherapy was set as one cycle.

The cost of supportive therapy for adverse events was determined based on the incidences of adverse events reported in the TAGS and ATTRACTION-2 trials. The adverse events analysed herein served to satisfy the following three criteria: (i) grade 3 or higher adverse events that occurred in the TAGS and ATTRACTION-2 trials, (ii) adverse events with an incidence of 2% or higher in the TAGS and ATTRACTION-2 trials and (iii) adverse events described in the package inserts of the drugs in Japan. The costs of supportive therapy for adverse events that satisfied these requirements were determined after a review by a medical expert. The expected costs of treatments with FTD/TPI and nivolumab were estimated to be JPY 17 183.4 (USD 156) and JPY 6071.1 (USD 55), respectively.

The cost of end-of-life care was set as JPY 1 120 000 (USD 10 182) based on the estimates used by the Ministry of Health, Labor and Welfare (19).

**Figure 2.**
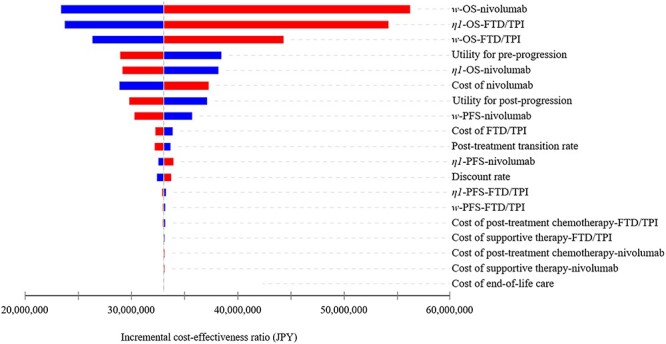
One-way sensitivity analysis. The tornado plot shows the incremental cost-effectiveness ratio for different model input parameters. Red bars represent an increase in value and blue bars represent a decrease in value. FTD/TPI, trifluridine/tipiracil; JPY, Japanese yen; OS, overall survival; PFS, progression-free survival.

A 2% discount was applied to the costs and effects as per Japanese guidelines by the Center for Outcomes Research and Economic Evaluation for Health (20).

### Sensitivity analysis

A sensitivity analysis was conducted to overcome uncertainties and assumptions in the data sources. A one-way sensitivity analysis was carried out to investigate the effect of each parameter used in the analysis. The costs of FTD/TPI and nivolumab varied by ±10%, whereas the QoL score, cost of supportive therapy and cost of end-of-life care varied by ±25%. The post-treatment transition rate varied within a range of 0–61%, based on the overall results of the TAGS and ATTRACTION-2 trials and the results for the Japanese subgroups. The discount varied within a range of 0–4% in accordance with the Japanese guidelines of the Center for Outcomes Research and Economic Evaluation for Health (20). The *η1* and *w* parameters in S(x) varied by two standard deviations for OS and PFS. Gamma distribution was used for estimating the costs of the drugs, supportive therapy and end-of-life care, whereas normal distribution was used for estimating the QoL score, *η1* and *w* parameters, and post-treatment transition rate. The factors used in the one-way sensitivity analysis, except for the discount rate, were randomly changed in the probabilistic sensitivity analysis. The details of the factors are shown in Table 2. Furthermore, 10 000 Monte Carlo simulations were conducted.

### Scenario analysis

In addition to nivolumab and FTD/TPI, irinotecan or docetaxel is used in patients with heavily pretreated metastatic gastric cancer in Japan. Therefore, a scenario analysis was carried out. During the post-treatment period, patients in the nivolumab arm were equally administered irinotecan, docetaxel or FTD/TPI and those in the FTD/TPI arm were administered irinotecan, docetaxel or nivolumab.

## Results

### Base-case results

The results of the base-case model are listed in Table 3. The expected median PFS was 1.99 and 1.55 months for FTD/TPI and nivolumab, respectively. The median OS was 5.59 and 5.26 months for FTD/TPI and nivolumab, respectively. The QALYs were 0.4379 and 0.5295 for FTD/TPI and nivolumab, respectively. The expected costs per patient were JPY 2 054 625 (USD 18 678) and JPY 5 018 148 (USD 45 620) for FTD/TPI and nivolumab, respectively, with a cost difference of JPY 2 963 523 (USD 26 941). The incremental cost-effectiveness ratio (ICER) of nivolumab versus FTD/TPI was JPY 32 352 489 (USD 294 113) per QALY gained [threshold value = JPY 7 500 000 (USD 68 182)].

**Figure 3.**
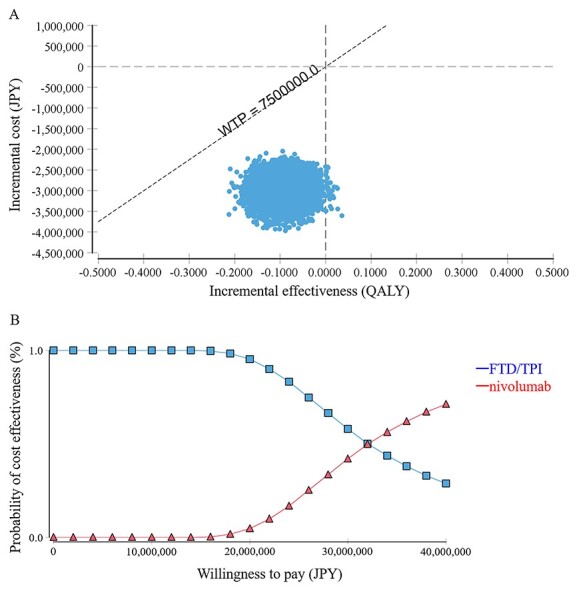
Probabilistic sensitivity analysis. (A) Scatter plot with 10 000 Monte Carlo simulations in the cost-effectiveness plane. (B) Cost-effectiveness acceptability curve representing the probability of the incremental cost-effectiveness ratio. FTD/TPI, trifluridine/tipiracil; JPY, Japanese yen; QALY, quality-adjusted life-year; WTP, willingness to pay.

### Sensitivity analysis

The results of the one-way sensitivity analysis are expressed as a tornado diagram in Fig. 2. The parameters affected by the ICER were ‘*w*-OS-nivolumab,’ ‘*η1*-OS-FTD/TPI,’ ‘*w*-OS-FTD/TPI,’ pre-progression QoL score, ‘*η1*-OS-nivolumab’ and the cost of nivolumab. The upper and lower limits of the ICER when each parameter was varied were JPY 56 247 817 (USD 511 344) and JPY 23 391 012 (USD 212 646), respectively. The results of the probabilistic sensitivity analyses are summarized in Fig. 3. The results of the cost-effectiveness acceptability curve indicated that the probability that the ICER for nivolumab versus FTD/TPI would fall below JPY 7 500 000 (USD 68 182)/QALY was 0%. The threshold for the ICER to reverse the cost-effectiveness of nivolumab and FTD/TPI was approximately JPY 32 500 000 (USD 295 455)/QALY.

### Scenario analysis

In the scenario analysis, the case changed the post-treatment and the expected costs per patient were JPY 1 986 721 (USD 18 061) and JPY 4 990 329 (USD 45 367) for FTD/TPI and nivolumab, respectively, with a difference of JPY 3 003 608 (USD 27 306). The ICER of nivolumab versus FTD/TPI was JPY 32 790 090 (USD 298 092)/QALY [threshold value = JPY 7 500 000 (USD 68 182)].

## Discussion

Nivolumab and FTD/TPI both revealed promising efficacy and tolerable safety profiles in patients with heavily pretreated metastatic gastric cancer. However, the cost-effectiveness of each drug has remained unclear. To our knowledge, our study is the first cost-effectiveness analysis of FTD/TPI versus nivolumab for patients with heavily pretreated metastatic gastric cancer. From the perspective of the Japanese public health care payer, FTD/TPI is more cost-effective than nivolumab for patients with heavily pretreated metastatic gastric cancer.

A cost-effectiveness evaluation system, similar to the systems used in the UK and other countries, was introduced in Japan in April 2019. Unlike in other countries, the system in Japan is used to adjust prices rather than determine the reimbursement of drugs and medical devices. This system revealed the stepwise price adjustments of the ICER value at JPY 5 000 000 (USD 45 455), JPY 7 500 000 (USD 68 182) and JPY 10 000 000 (USD 90 909) (20). In particular, the ICER values of anticancer drugs underwent the stepwise price adjustments at JPY 7 500 000 (USD 68 182), JPY 11 250 000 (USD 102 273) and JPY 15 000 000 (USD 136 364). In this study, we determined the expected median PFS and expected median OS, which were 1.99 and 5.59 months for FTD/TPI and 1.55 and 5.26 months for nivolumab, respectively. The median PFS and median OS were similar in both treatment groups. Compared with that for nivolumab, the expected QALY for FTD/TPI was lower by 0.0916, and the expected cost was lower by approximately JPY 3 000 000 (USD 27 273). This slight difference in the expected QALY may have been due to the presence of long-term survivors in the nivolumab group in the ATTRACTION-2 trial.

Considering that the expected QALY was higher in the nivolumab group than in the FTD/TPI group, we determined the ICER of nivolumab to FTD/TPI. A single technology appraisal of cladribine for treating patients with relapsing–remitting multiple sclerosis has suggested that a new drug that is less effective and less costly than an existing drug is considered cost-effective if the ICER generated is above the level considered acceptable (21); the reduced efficacy of a new drug relative to its competitor has been previously reported in the UK during NICE technology assessment. Per this recommendation, FTD/TPI can be considered cost-effective compared to nivolumab. The expected median PFS and OS were equivalent between the FTD/TPI and nivolumab groups, whereas the QALY for FTD/TPI was slightly lower (by 0.0916) than that for nivolumab; however, the cost was greatly reduced for FTD/TPI. Furthermore, the sensitivity analysis revealed that nivolumab was less likely to be cost-effective than FTD/TPI. The probability of nivolumab being cost-effective relative to FTD/TPI [ICER below JPY 7 500 000 (USD 68 182)] was 0%. Nivolumab would only be more cost-effective than FTD/TPI if WTP exceeded approximately JPY 32 500 000 (USD 295 455)/QALY.

This study has some limitations. First, the study used an indirect comparison method. Because there were no head-to-head randomized controlled trials for comparison between FTD/TPI and nivolumab, the efficacy and safety data were derived from individual trials. However, the sensitivity analysis revealed similar results for varying efficacy parameters; thus, it was unlikely that the indirect comparison affected the results. Furthermore, the estimated median PFS and OS were similar to the actual median PFS and OS in the TAGS and ATTRACTION-2 trials. Second, because EQ-5D data were not collected in the TAGS and ATTRACTION-2 trials, the QoL score was derived from the INTEGRATE trial. The INTEGRATE trial has a close patient background to the TAGS and ATTRACTION-2 trials. However, there were some differences, such as in terms of treatment line and participating countries. For example, all patients received two or more lines of chemotherapy in the TAGS and ATTRACTION-2 trials, whereas 58% of patients received two or more lines of chemotherapy in the INTEGRATE trial (17); the derived QoL score might be better than the actual QoL score of patients who received two or more lines of chemotherapy. Moreover, the INTEGRATE trial was conducted in Australia, New Zealand, South Korea and Canada, in other words, no Japanese patients were enrolled in the INTEGRATE trial. However, from a clinical perspective, such as an incidence of an adverse event, it is unlikely that FTD/TPI and nivolumab would show a significant difference in QoL score. It may be interesting to conduct an analysis using EQ-5D data from post-marketing surveillance of each drug. Third, the costs were based on assumptions rather than on actual figures; however, the assumptions were based on the treatment policies adopted in real-world clinical practice. Therefore, at the very least, it can be expected that the results did not favour one group over the other. Moreover, because the sensitivity analysis revealed that the costs of the drugs did not greatly affect the ICER, the assumptions were not considered a problem. Finally, as costs and insurance systems vary by country, the applicability of our results to other countries is limited. Our results would be most helpful to clinicians and payers in countries in which nivolumab is reimbursed for the treatment of metastatic gastric cancer.

In addition to the efficacy and safety, our cost-effectiveness analysis showed the additional value of FTD/TPI. The efficiency data of FTD/TPI, produced by our analysis, could be somewhat helpful for decision makers, such as clinicians and payers. In conclusion, from the perspective of the Japanese public health care payer, FTD/TPI was shown to be more cost-effective than nivolumab for patients with heavily pretreated gastric cancer.
